# Association of reduced DTI-ALPS index and increased free water with glymphatic system alterations in noise-induced hearing loss: a neuroimaging study

**DOI:** 10.1038/s41598-025-32299-x

**Published:** 2025-12-11

**Authors:** Liping Wang, Wei Lian, Ranran Huang, Aijie Wang, Minghui Lv, Xinyao Zhao, Zengcai Zhang, Guowei Zhang

**Affiliations:** 1 Imaging Department, Yantaishan Hospital, Yantai, China; 2 Shandong Luhang Intelligent Technology Co., LTD, Yantai, China

**Keywords:** Noise-induced hearing loss, Diffusion tensor imaging along perivascular spaces, Free water, Glymphatic system, Biomarkers, Diseases, Medical research, Neurology, Neuroscience

## Abstract

To investigate diffusion changes suggestive of possible glymphatic alterations in patients with noise-induced hearing loss (NIHL), and to evaluate their relationships with free water (FW) and clinical characteristics. DTI scans were acquired from 48 NIHL patients and 40 healthy controls (HCs). The diffusion tensor imaging along perivascular spaces (DTI-ALPS) technique and FW diffusion model were used to calculate DTI-ALPS and FW values. Between-group differences were assessed using ANCOVA. Spearman correlation analysis was conducted between DTI-ALPS, FW, Hamilton Anxiety Scale (HAMA), and better-ear monaural threshold weighted value (MTWV). Compared with HCs, NIHL patients exhibited significantly reduced DTI-ALPS indices (mean: *β* = – 0.073, *p* = 0.008; left: *β* = – 0.081, *p* = 0.006; right: *β* = – 0.068, *p* = 0.026) and increased FW (*β* = 0.017, *p* = 0.019). Within the NIHL group, the left DTI-ALPS index was significantly lower than the right DTI-ALPS index (*p* < 0.001). Significant negative correlations were observed: mean DTI-ALPS with HAMA (*r* = – 0.406, *p* = 0.004) and FW (*r* = – 0.510, *p* < 0.001); left DTI-ALPS with HAMA (*r* = – 0.463, *p* < 0.001) and FW (*r* = – 0.459, *p* = 0.001); and right DTI-ALPS with FW (*r* = – 0.488, *p* < 0.001). All above results remained significant after FDR correction. Correlations of DTI-ALPS with MTWV (mean: *r* = – 0.311, *p* = 0.031; left: *r* = – 0.295, *p* = 0.042; right: *r* = – 0.287, *p* = 0.048) and right DTI-ALPS with HAMA (*r* = -0.308, *p* = 0.033) did not survive FDR correction. Patients with NIHL exhibited altered diffusion patterns suggestive of possible glymphatic alterations. These cross-sectional, correlational findings do not permit causal inferences. Longitudinal studies in larger and more diverse cohorts are needed for validation.

## Introduction

Noise-induced hearing loss (NIHL), as a kind of sensorineural hearing impairment, mainly results from chronic exposure to high-noise settings like workplaces and daily environments^1^. As one of the most prevalent occupational diseases globally, NIHL has emerged as a significant public health concern requiring urgent attention^2–4^. It not only causes multiple pathological changes in the auditory system’s physiological structure but also leads to various neurological and psychological consequences, including depression, anxiety disorders, and cognitive dysfunction^5,6^. Consequently, in-depth exploration of the pathological mechanisms in the central nervous system of NIHL has significant practical importance for early warning, intervention and prognosis assessment.

The central nervous system possesses a specialized metabolic waste clearance system analogous to the glymphatic system, which plays a crucial role in removing cerebral metabolic byproducts^7^. This system facilitates transport and clearance of metabolic products through flow and exchange of cerebrospinal fluid and interstitial fluid within perivascular spaces^8^. Glymphatic system dysfunction causes accumulation of toxic wastes, including amyloid-β and tau proteins, resulting in a series of damage^9,10^. Some studies confirmed that glymphatic system dysfunction is strongly linked to the occurrence and development of various neurological diseases, including chronic tinnitus^11^, chronic migraine^12^, presbycusis^9^, frontotemporal dementia variants^13^, systemic lupus erythematosus^14^, Parkinson’s disease^15^, amyotrophic lateral sclerosis^16^, etc. So far, glymphatic system alterations in patients with NIHL remain unclear.

Diffusion tensor imaging (DTI), as a non-invasive magnetic resonance imaging technique, is able to reflect microstructural changes in brain tissue by detecting the directionality and magnitude of water-molecule diffusion^17^. DTI has been widely applied in the study of glymphatic system^12^. In particular, diffusion tensor imaging along perivascular spaces (DTI-ALPS)^18^ and the free water (FW) diffusion model based on bi-tensor DTI^19^ have become widely used indirect, non-invasive proxies for investigating glymphatic system function^10,12^. Andica et al. discovered lower DTI-ALPS indices but higher FW in elderly metabolic syndrome patients, and a significant correlation between higher FW and lower DTI-ALPS indices^10^. In the study on obstructive sleep apnea, severe patients exhibited higher mean free water. The DTI-ALPS of patient group was lower than healthy controls group, and correlated with cognitive performance^20^. Li et al. discovered that decreased DTI-ALPS and increased FW fraction were related to the progression of MRI markers in cerebral small vessel disease, along with cognitive decline related to the disease^21^.

Some evidence indicates that chronic noise exposure induces central nervous system changes beyond the auditory pathway, including structural and functional brain alterations and increased anxiety^22,23^. Recent studies have reported diffusion changes suggestive of possible glymphatic alterations in presbycusis^9^, congenital sensorineural hearing loss^24^, and chronic tinnitus^11^. We therefore hypothesized that NIHL, as an environmentally acquired condition, would also exhibit similar diffusion changes on indirect, non-quantitative proxies of glymphatic alterations.

In the presnet study, the DTI-ALPS index and the FW diffusion model are employed to explore whether such changes are present in NIHL patients and whether they are associated with FW values, clinical characteristics.

## Materials and methods

### Subjects

According to the national standard of the People’s Republic of China, GBZ 49-2014 “Diagnosis of Occupational Noise Deafness”, 48 NIHL patients were recruited as research subjects from the occupational department of Yantaishan Hospital between 2014 and 2020. The NIHL group consisted predominantly of individuals engaged in rock drilling and welding. As a result, all participants were adult males. Meanwhile, 40 healthy controls (HCs) matched to the NIHL group for age, education level, and gender were enrolled in the study. The clinical characteristics of all the participants, including age, education level, better-ear monaural threshold weighted value (MTWV), and Hamilton Anxiety Scale (HAMA) score, were collected and analyzed. The diagnosing criteria for all participants: for individuals with an average hearing threshold above 40 dB in both ears for high-frequency ranges (3000, 4000, and 6000 Hz), diagnosis and classification are based on the weighted values of the better whispered frequency (500, 1000, and 2000 Hz) and the hearing threshold at 4000 Hz. NIHL is diagnosed when MTWV is ≥ 26 dB; while MTWV below 25 dB was diagnosed as normal^23^. All patients met the diagnostic criterion of MTWV ≥ 26 dB HL. All HCs had normal hearing defined as MTWV < 25 dB HL. By study design, only the binary inclusion criterion (normal hearing: yes/no) was recorded at the time of recruitment; exact MTWV values are not available for the HCs. The inclusion and exclusion criteria for all participants: adult Han Chinese males (due to the male-dominated nature of the occupations involved); educational level: primary school or above; participants had no personal/family history of psychiatric or neurological disorders and did not use psychotropic drugs.

The study was approved by the Ethics Committee of Yantaishan Hospital (Ethics Approval No.: Yanshanlun 2023014). All participants provided written informed consent. The entire study was conducted in strict accordance with the Declaration of Helsinki and relevant medical ethical norms to ensure the rights and interests of participants as well as data security.

### Imaging acquisition and preprocessing

#### Imaging acquisition

The GE Discovery MR 750 3.0T with 8-channel brain coil was used for scanning all participants. The DTI sequence parameters included: TR = 5500ms, minimum TE, slice thickness = 3.0 mm, gap = 0 mm, FOV = 24 cm × 24 cm, flip angle = 90°, matrix size = 128 × 128, NEX = 1, b-value = 0,1000s/mm², gradient direction = 50, scanning time = 4 min 46 s.

#### Imaging preprocessing

Diffusion MRI data were processed with FMRIB Software Library (FSL, http://www.fmrib.ox.ac.uk/fsl/) and MRtrix3 (https://www.mrtrix.org/). The preprocessing steps included denoising, Gibbs artifact correction, eddy current and motion correction, B1 field bias correction, and skull stripping.

### DTI-ALPS index calculation

The DTI-ALPS index was calculated with a validated semi-automated approach through bash scripts^10,25^. The procedure involved the following steps:

1. ROI definition. Spherical ROIs (5 mm diameter) were placed at the level of the lateral ventricles on projection and association fibers using the JHU-ICBM-FA template. The central coordinates of ROIs for bilateral projection fibers and association fibers were as follows: L_proj (116, 110, 99), L_assoc (128, 110, 99), R_proj (64, 110, 99), and R_assoc (51, 110, 99).
2. Images processing and registration. FA and tensor maps were generated with FSL. Individual FA maps were registered to the JHU-ICBM-FA template. The resulting transformation matrix was subsequently applied to align the corresponding tensor map to the template space. All registered images were manually inspected to ensure quality.
3. Diffusion values extraction. The diffusion components (Dxx, Dyy, Dzz) were extracted from the registered tensor maps, and their diffusion values were obtained within the predefined ROIs using the following variable conventions:

Dxxproj_L, Dxxproj_R: X-axis diffusivity in left/right projection fibers.

Dxxassoc_L, Dxxassoc_R: X-axis diffusivity in left/right association fibers.

Dyyproj_L, Dyyproj_R: Y-axis diffusivity in left/right projection fibers.

Dzzassoc_L, Dzzassoc_R: Z-axis diffusivity in left/right association fibers.

(4) DTI-ALPS index calculation. The left, right, and mean DTI-ALPS indices were calculated in the following manner:

Left DTI-ALPS = ((Dxxproj_L + Dxxassoc_L)/2) / ((Dyyproj_L + Dzzassoc_L)/2).

Right DTI-ALPS = ((Dxxproj_R + Dxxassoc_R)/2) / ((Dyyproj_R + Dzzassoc_R)/2).

Mean DTI-ALPS = ((Dxxproj_L + Dxxassoc_L + Dxxproj_R + Dxxassoc_R)/4) / ((Dyyproj_L + Dzzassoc_L + Dyyproj_R + Dzzassoc_R)/4).

### FW in white matter calculation

As described in previous studies^10,26^, the regularized bi-tensor model was used to construct FW maps, and Tract-Based Spatial Statistics (TBSS)^27^was employed to obtain FW values along the white matter skeleton. The workflow was as follows:

1. Generate free water maps by fitting a regularized bi-tensor model using the Diffusion Imaging (Dipy) in Python open-source package^28^.
2. Using TBSS, create the group-level mean FA map from all subjects first, then extract FA skeleton map with an FA threshold level of 0.2.
3. Project the FW map of each participant onto the mean FA skeleton map to obtain its FW map on the white matter skeleton.
4. Extract FW values based on the skeletonized FW maps.

### Statistical analysis

Statistical analyses were performed in R software (version 4.3.3; https://www.r-project.org/). The normality of continuous variables was evaluated via Shapiro-Wilk test. Independent sample t-tests or Mann-Whitney U tests were used to analyze group differences in demographic and clinical characteristics.

After controlling for age and education level, analysis of covariance (ANCOVA) was used to examine between-group differences in mean DTI-ALPS, left DTI-ALPS, right DTI-ALPS, and FW. Prior to the ANCOVA, the model assumptions were tested for normality of residuals and homoscedasticity using the Shapiro-Wilk test and Levene’s test, respectively. Both assumptions were met. Paired t-tests were applied to assess within-group differences in bilateral hemispheric DTI-ALPS within both the NIHL group and the HCs group.

Spearman correlation analysis was employed to explore the correlations between DTI-ALPS and clinical characteristics as well as FW. A value of *p* < 0.05 was considered statistically significant and Benjamini–Hochberg FDR correction was used for multiple comparisons.

## Results

### Demographic and clinical characteristics

As detailed in Table 1, there were no significant differences between the two groups for age (*p* = 0.516), education level (*p* = 0.156). The HAMA score (*p* < 0.001) was significantly higher in the NIHL group than in the HCs group.

**Table 1:** Demographic and clinical characteristics of participants.

Characteristic	NIHL(*n* = 48)	HCs(*n* = 40)	t	*p*
Age(years)	46.04 ± 7.18	47.10 ± 8.05	– 0.652	0.516
Education level(years)	11.60 ± 2.08	12.45 ± 3.21	– 1.434	0.156
HAMA score	6.42 ± 3.89	3.60 ± 0.95	4.469	< 0.001***
MTWV (db)	43.45 ± 13.31	N/a	N/a	N/a

NIHL: noise-induced hearing loss; HCs: healthy controls; HAMA: Hamilton Anxiety Scale; MTWV: better-ear monaural threshold weighted value. Exact MTWV values for HCs were not recorded (normal hearing defined as better-ear MTWV < 25 dB HL per GBZ 49-2014). Significance: *p < 0.05, **p < 0.01, ***p < 0.001.

### Between-group differences in DTI-ALPS indices and FW

**Fig. 1:**
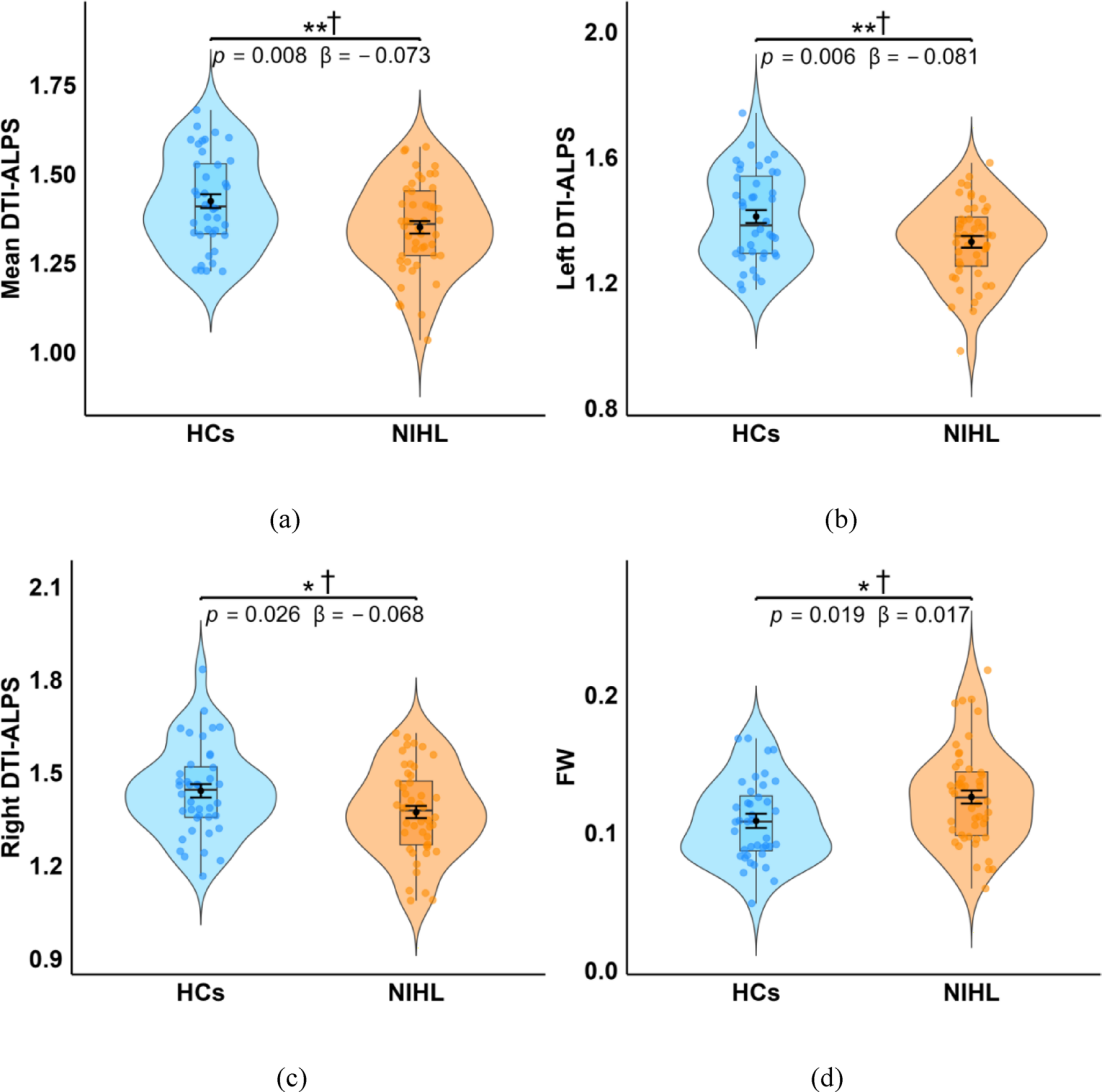
Comparison of DTI-ALPS indices and FW between the NIHL and HCs groups. The DTI-ALPS (**a**, **b**, **c**) and FW (d) are presented through violin plots with embedded box plots. Black diamonds: least-squares mean ± SE from ANCOVA controlling for age and education level. Significance: *p < 0.05, **p < 0.01, ***p < 0.001 (all uncorrected); †*p* < 0.05 (FDR-corrected).

Significant intergroup differences in DTI-ALPS indices and FW between the NIHL and HCs groups are presented in Table 2; Fig. 1. After adjusting for age and education level, the mean DTI-ALPS index (*β* = -0.073, *p* = 0.008), left DTI-ALPS index (*β* = -0.081, *p* = 0.006) and right DTI-ALPS index (*β* = -0.068, *p* = 0.026) were significantly lower in the NIHL group than in the HCs group, and all survived FDR correction. Conversely, FW values (*β* = 0.017, *p* = 0.019) were significantly higher in the NIHL group, and remained significant after FDR correction.

**Table 2:** Between-group differences in DTI-ALPS and FW.

Characteristic	Group	Mean (SD)	Adj. Mean (SE)	β(95% CI)	F	*P*	η²*p*
DTI-ALPS							
Mean	NIHL	1.352(0.128)	1.352(0.018)	– 0.073 (– 0.126, – 0.02)	7.407	0.008**†	0.081
	HCs	1.423(0.132)	1.425(0.021)				
Left	NIHL	1.331(0.126)	1.331(0.018)	– 0.081 (– 0.137, – 0.024)	7.852	0.006**†	0.085
	HCs	1.409(0.145)	1.411(0.023)				
Right	NIHL	1.374(0.143)	1.372(0.021)	– 0.068 (– 0.128, – 0.009)	5.172	0.026*†	0.058
	HCs	1.440(0.146)	1.440(0.023)				
FW	NIHL	0.126(0.036)	0.126(0.005)	0.017 (0.003, 0.032)	5.681	0.019*†	0.063
	HCs	0.108(0.029)	0.108(0.005)				

NIHL: noise-induced hearing loss; HCs: healthy controls; DTI-ALPS: diffusion tensor imaging along perivascular space; FW: free water. Raw data: Mean (SD) = arithmetic mean ± standard deviation. Model estimates: Adj. Mean (SE) = least-squares mean ± standard error from ANCOVA controlling for age and education level. *β* = adjusted mean difference (NIHL minus HCs). 95%CI = two-sided confidence interval. *η²p* = effect sizes. Significance: *p < 0.05, **p < 0.01, ***p < 0.001 (all uncorrected); †*p* < 0.05 (FDR-corrected).

### Within-group inter-hemispheric differences in DTI-ALPS indices

**Fig. 2:**
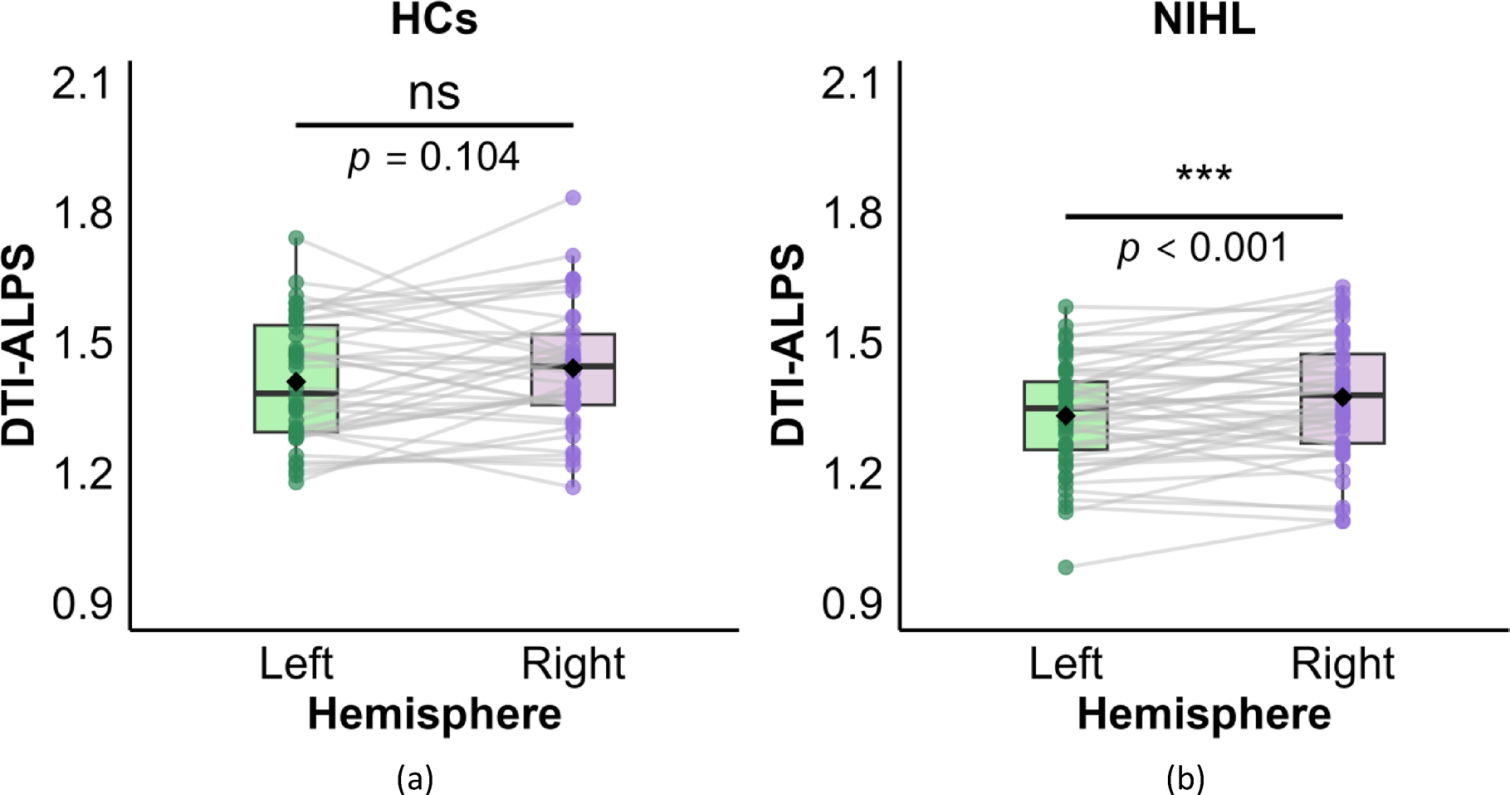
Within-group differences in DTI-ALPS indices between left and right hemispheres in the HCs (**a**) and NIHL (**b**) groups. The DTI-ALPS indices are presented through violin plots with embedded box plots. Gray lines: within-subject hemispheric connections. Black diamonds: group means. Significance: *p < 0.05, **p < 0.01, ***p < 0.001.

Within-group differences in DTI-ALPS indices between left and right hemispheres are presented in Table 3, Fig. 2. A significant inter-hemispheric difference was observed only in the NIHL group, where the left DTI-ALPS index was significantly lower than the right DTI-ALPS index (*p* < 0.001). Conversely, no significant inter-hemispheric difference (*p* = 0.104) was found in the HCs group.

**Table 3:** Inter-hemispheric differences in DTI-ALPS.

Groups	Left DTI-ALPS	Right DTI-ALPS	t	*p*
NIHL	1.331 ± 0.126	1.374 ± 0.143	-3.534	< 0.001***
HCs	1.409 ± 0.145	1.440 ± 0.146	-1.666	0.104

NIHL: noise-induced hearing loss; HCs: healthy controls; DTI-ALPS: diffusion tensor imaging along perivascular space. Significance: *p < 0.05, **p < 0.01, ***p < 0.001.

### Correlations of DTI‑ALPS with clinical characteristics and FW

**Fig. 3:**
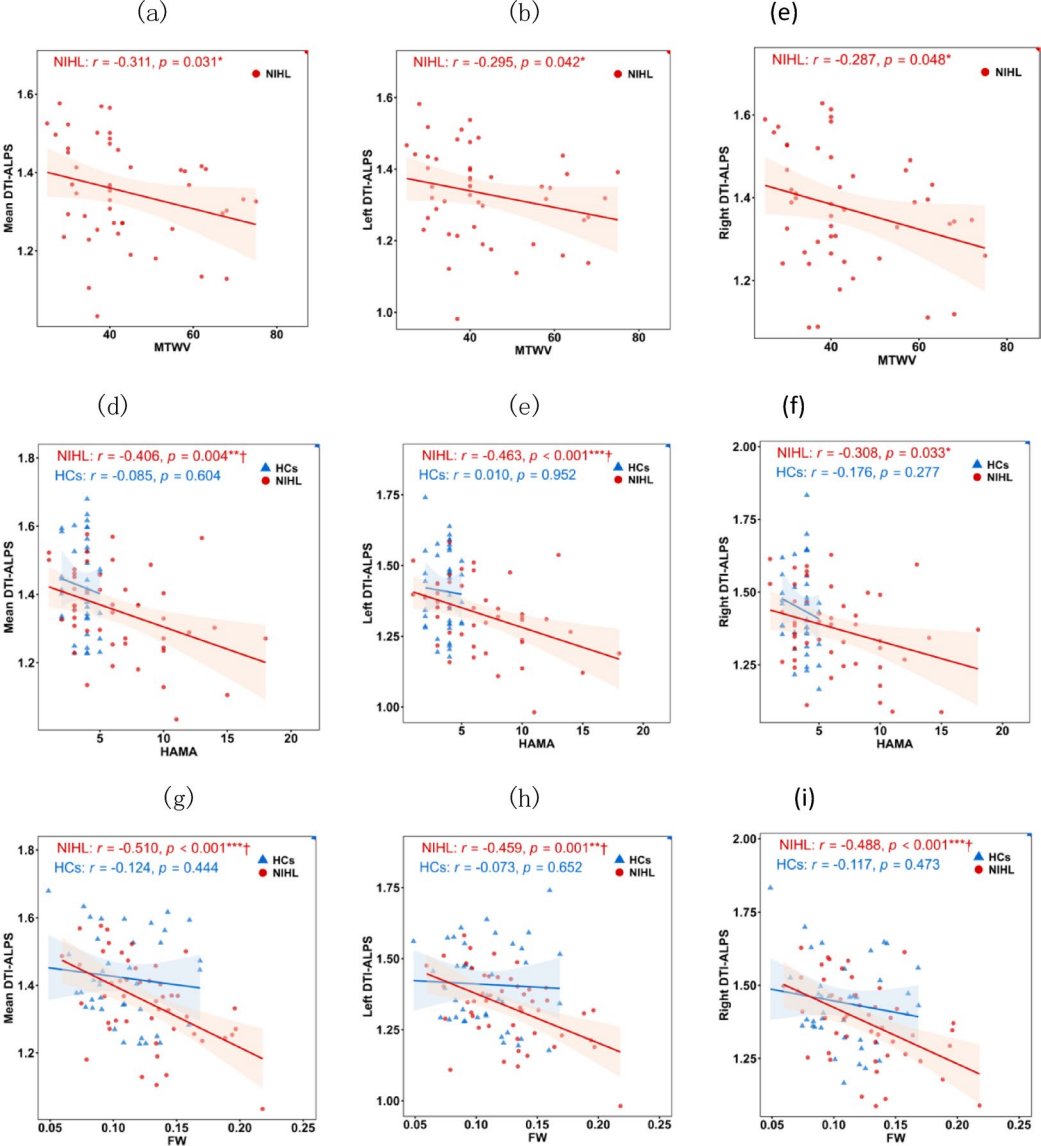
Correlations of DTI‑ALPS indices with clinical characteristics and FW in the NIHL (red) and HCs (blue) groups. Graphs (**a**, **b**,**c**) show the correlations of DTI-ALPS indices and MTWV; graphs (**d**, **e**,**f**) show the correlations of DTI-ALPS indices and HAMA; graphs (**g**, **h**,**i**) show the correlations of DTI-ALPS indices and FW. Significance: *p < 0.05, **p < 0.01, ***p < 0.001(all uncorrected); †*p* < 0.05 (FDR-corrected).

The correlations between DTI-ALPS indices and clinical characteristics are shown in Table 4; Fig. 3. In the NIHL group, the mean DTI-ALPS showed significant negative correlations with MTWV (*r* = -0.311, *p* = 0.031), HAMA (*r* = – 0.406, *p* = 0.004), and FW (*r* = -0.510, *p* < 0.001). Similarly, the left DTI-ALPS was negatively correlated with MTWV (*r* = -0.295, *p* = 0.042), HAMA (*r* = -0.463, *p* < 0.001) and FW (*r* = -0.459, *p* = 0.001). The right DTI-ALPS was also negatively correlated with MTWV (*r* = -0.287, *p* = 0.048), HAMA (*r* = -0.308, *p* = 0.033) and FW (*r* = -0.488, *p* < 0.001). After FDR correction, the negative correlations of all three DTI-ALPS indices with FW, as well as both the mean and left DTI-ALPS with HAMA, remained significant. In contrast, the correlations involving all three DTI-ALPS indices with MTWV and right DTI-ALPS with HAMA did not survive FDR correlation. Furthermore, no significant correlations were observed between any DTI-ALPS indices and clinical variables or FW in the HCs group.

**Table 4:** Correlations of DTI‑ALPS with clinical measures and FW.

Characteristic	MTWV	HAMA	FW
	*r* *p*	*r* *p*	*r* *p*
NIHL			
Mean DTI-ALPS	– 0.311 0.031*	– 0.406 0.004**†	– 0.510 <0.001***†
Left DTI-ALPS	– 0.295 0.042*	– 0.463 <0.001***†	– 0.459 0.001**†
Right DTI-ALPS	– 0.287 0.048*	– 0.308 0.033*	– 0.488 <0.001***†
HCs			
Mean DTI-ALPS	N/a N/a	– 0.085 0.604	– 0.124 0.444
Left DTI-ALPS	N/a N/a	– 0.010 0.952	– 0.073 0.652
Right DTI-ALPS	N/a N/a	– 0.176 0.277	– 0.117 0.473

NIHL: noise-induced hearing loss; HCs: healthy controls; HAMA: Hamilton Anxiety Scale; MTWV: better-ear monaural threshold weighted value; DTI-ALPS: diffusion tensor imaging along perivascular space; FW: free water. Significance: *p < 0.05, **p < 0.01, ***p < 0.001(all uncorrected); †*p* < 0.05 (FDR-corrected).

## Discussion

This study used non-invasive DTI-ALPS technique and FW diffusion model to assess glymphatic system status in patients with NIHL. We observed diffusion changes suggestive of possible glymphatic alterations in the NIHL group. These findings were specifically manifested as: (1) Compared to the HCs, the DTI-ALPS indices of left hemisphere, right hemisphere, and whole brain in NIHL patients were significantly decreased, while the FW values were significantly increased. (2) Significant inter-hemispheric differences emerged in DTI-ALPS indices in NIHL patients, and the left hemisphere DTI-ALPS index was lower. (3) The correlation analysis showed that the DTI-ALPS indices in the NIHL group were significantly negatively correlated with FW values, HAMA scores, and MTWV scores, whereas no significant relationships were observed in the HCs group.

### DTI-ALPS analysis

The diffusion tensor imaging along perivascular space (DTI-ALPS) index is a non-invasive, indirect, and non-quantitative MRI measure used to indirectly evaluate the glymphatic clearance efficiency. A higher ALPS index reflects greater diffusion efficiency along these spaces, which is thought to correspond to stronger glymphatic clearance capacity^18^. Conversely, an ALPS index closer to 1 indicates more significant restriction of diffusion perpendicular to the vessels, corresponding to lower effective diffusivity along the perivascular spaces and suggestive of impaired clearance. In this study, it was found that DTI-ALPS indices in the left hemisphere, right hemisphere, and whole brain were significantly lower in NIHL patients than those in the HCs. These findings indicate diffusion changes suggestive of possible glymphatic system alterations in NIHL patients. Prior studies have reported reduced DTI-ALPS indices in presbycusis^9^, congenital sensorineural hearing loss^24^, and chronic tinnitus^11^. Our findings extend these observations to NIHL, an acquired auditory disorder driven primarily by environmental noise exposure rather than aging or isolated tinnitus. This suggests that altered diffusion patterns consistent with possible glymphatic changes may occur across diverse forms of peripheral auditory damage. Furthermore, significantly lower average, right, and left ALPS indices have also been reported in former professional athletes^29^ and patients with alcohol use disorder^30^. The discovery suggests that diffusion changes suggestive of possible glymphatic alterations may represent one of the central imaging changes associated with progression from peripheral noise-induced damage to the central nervous system.

Most previous DTI-ALPS studies have focused on between-group comparisons, whereas within-subject left–right differences have received less attention. In the present study, a significant hemispheric difference in DTI-ALPS index was observed exclusively in the NIHL group, with lower values in the left hemisphere compared with the right hemisphere. No significant left–right difference was found in HCs. In studies of other neurodegenerative diseases, it was also found that the glymphatic circulation alterations exhibited asymmetry in the cerebral hemispheres. Shen et al. found that the glymphatic system changes existed in both hemispheres of patients with PD, and the alterations in the left hemisphere were more significant^15^. It was further confirmed by Meng et al. that the left DTI-ALPS index was lower than the right DTI-ALPS index in PD patients^31^. At present, the mechanisms and clinical significance of this asymmetry in NIHL remain unclear. Although all participants were right-handed and the left hemisphere is known to dominate auditory-language processing and to exhibit higher basal metabolism^32^, any causal link between these factors and the observed left-sided reduction in DTI-ALPS index is speculative. Further studies, particularly longitudinal designs, are needed to determine whether this asymmetry reflects differential vulnerability of the dominant hemisphere to noise-related injury.

### FW analysis

FW is defined as water molecules that are not restricted by biological membranes and can freely diffuse. In brain tissue, the cerebrospinal fluid within the ventricular system and the free fluid in the parenchymal spaces both belong to free water. Based on the dual-tensor model of DTI, the volume fraction of FW can be accurately quantified by separating the free diffusion and restricted diffusion components within voxels^33^. In recent years, the FW has become a crucial imaging biomarker for the pathological progression of neurological diseases. Neuroimaging studies had demonstrated that extensive loss of white matter volume could increase interstitial space and lead to an increase in the fluid content in the brain, while FW had become an important indicator for quantifying this fluid content^34^. Significant increase of FW had been observed in various neurological diseases, including traumatic brain injury^34^, idiopathic REM sleep behavior disorder^35^, schizophrenia^26^, and Parkinson’s disease^36^. FW has become a potential and indirect imaging biomarker which may reflect the central pathological processes of diseases such as neuroinflammation, cerebral edema, and atrophy.

Compared to the HCs group, FW values were significantly higher in the NIHL group and negatively correlated with DTI-ALPS indices. This pattern is consistent with observations in other neurological disorders^34–36^. This suggests that cerebral fluid metabolism may also be changed in patients with NIHL. The concurrent decrease in DTI-ALPS index and increase in FW indicates suggests altered perivascular and interstitial fluid dynamics in NIHL. Although a vicious cycle of reduced clearance leading to greater fluid retention (and vice versa) is biologically plausible, direct evidence for such a feedback loop in NIHL is currently lacking and remains speculative. Future mechanistic and longitudinal studies are needed to confirm the pathophysiological relationship between these imaging markers and central auditory dysfunction.

### Correlation analysis

In addition, the mean DTI-ALPS index in patients with NIHL were significantly negatively correlated with MTWV, HAMA scores, and FW, whereas these significant associations were not observed in HCs. These findings are consistent with previous reports of a negative correlation between the DTI-ALPS index and Tinnitus Handicap Inventory^37^, as well as FW with DTI-ALPS^10^. Collectively, these results indicate that diffusion changes suggestive of possible glymphatic alterations in patients with NIHL are associated with greater MTWV, higher HAMA, and higher FW. These associations extend previously reported links between DTI-ALPS and symptom severity in tinnitus^37^ to NIHL.

### Limitations

Several limitations should be acknowledged. First, the cross-sectional design prevents any causal or temporal inferences. Second, the study included only adult male participants, which limits the generalizability of our findings to females; potential sex differences in glymphatic function or susceptibility to noise-induced damage could not be evaluated. Third, although the total sample size is reasonable for an exploratory study, it remains relatively modest and may have reduced statistical power for detecting subtler effects. Future studies should employ longitudinal designs with larger and sex-balanced cohorts to confirm the present observations.

## Conclusion

Our findings demonstrate altered diffusion patterns suggestive of possible glymphatic alterations in patients with NIHL, indicating potential disruption in perivascular fluid regulation. These alterations were manifested as significantly reduced DTI-ALPS indices and elevated FW values compared with HCs. However, the cross-sectional design, exclusive inclusion of male participants, and relatively modest sample size preclude any causal inferences or generalization of these observations. In future, longitudinal studies in larger and sex-balanced cohorts are required to validate these preliminary findings and to clarify the temporal and mechanistic role of glymphatic alterations.

## Data Availability

Due to privacy or ethical restrictions, the data of this study are not publicly available. But data are available upon reasonable request from the corresponding author.
